# Non-Invasive *In Vivo* Imaging of Calcium Signaling in Mice

**DOI:** 10.1371/journal.pone.0000974

**Published:** 2007-10-03

**Authors:** Kelly L. Rogers, Sandrine Picaud, Emilie Roncali, Raphaël Boisgard, Cesare Colasante, Jacques Stinnakre, Bertrand Tavitian, Philippe Brûlet

**Affiliations:** 1 Unité d'Embryologie Moléculaire, CNRS URA 2578, Institut Pasteur, Paris, France; 2 CEA, Service Hospitalier Frédéric Joliot, Inserm, U 803, Imagerie de l'expression des gènes, Orsay, France; California Institute of Technology, United States of America

## Abstract

Rapid and transient elevations of Ca^2+^ within cellular microdomains play a critical role in the regulation of many signal transduction pathways. Described here is a genetic approach for non-invasive detection of localized Ca^2+^ concentration ([Ca^2+^]) rises in live animals using bioluminescence imaging (BLI). Transgenic mice conditionally expressing the Ca^2+^-sensitive bioluminescent reporter GFP-aequorin targeted to the mitochondrial matrix were studied in several experimental paradigms. Rapid [Ca^2+^] rises inside the mitochondrial matrix could be readily detected during single-twitch muscle contractions. Whole body patterns of [Ca^2+^] were monitored in freely moving mice and during epileptic seizures. Furthermore, variations in mitochondrial [Ca^2+^] correlated to behavioral components of the sleep/wake cycle were observed during prolonged whole body recordings of newborn mice. This non-invasive imaging technique opens new avenues for the analysis of Ca^2+^ signaling whenever whole body information in freely moving animals is desired, in particular during behavioral and developmental studies.

## Introduction

Calcium (Ca^2+^) is a universal second messenger that regulates cell signaling pathways involved in muscular contraction, hormone secretion, neurotransmitter release, cellular metabolism, apoptosis, etc. Abnormalities in the homeostatic regulation of Ca^2+^ signaling have pathological consequences relevant to many common diseases (e.g. Alzheimer's, diabetes, cancer, migraine, cardiovascular disorders) [1]–[4].

The selective regulation of many signal transduction pathways is partly facilitated by intracellular Ca^2+^ concentration ([Ca^2+^]) rises that are restricted in space (e.g. nano- & micro-domains), amplitude (100 nM–100's µM) and time (microseconds to seconds). The propagation of Ca^2+^ waves or other second messengers associated with Ca^2+^ signaling may also affect remote cellular regions, tissues, or other parts of an organism. In addition, Ca^2+^ oscillations of varying frequencies are important for gene expression and other rhythmic activities [1], [5].

In keeping with the versatile nature of Ca^2+^ signals (e.g. localization, amplitude, kinetics and frequency), optical imaging methods can provide the high degree of spatio-temporal resolution necessary for their characterization. Recently, these methods have been extended to *in vivo* approaches allowing [Ca^2+^] within the intact animal to be investigated under more physiological conditions [6]–[9]. Notably, *in vivo* imaging of the neonatal brain by fiber-optic based detection of Ca^2+^ sensitive dyes, led to the identification of early network Ca^2+^ oscillations (ENOs) occurring in the cortex of newborn mice during sleep [9]. In another approach, a genetically encoded Ca^2+^ sensitive probe was expressed in the muscles of live animals and gave accurate information about [Ca^2+^] in the mitochondrial matrix ([Ca^2+^]_m_) during relaxation/contraction cycles [8]. However, all of these methods are invasive and restricted to small fields of view (*ca.* 1 mm^2^), preventing longitudinal analyses or studies on Ca^2+^ signals over long distances and simultaneously across multiple systems.

Bioluminescent probes in which light is produced by enzymatic breakdown of a substrate have an excellent signal-to-noise ratio (*i.e.* background noise is limited to that of the light detector). In recent years, whole animal bioluminescence imaging (BLI) has emerged as a sensitive method for localizing gene expression or cell migration in live animals [10]–[12]. GFP-aequorin (GA) is a bioluminescent Ca^2+^-reporter, which is based on the light emitting system of the jellyfish, *Aequorea victoria* [13]. Upon Ca^2+^ binding, aequorin undergoes a conformational change that oxidizes its substrate coelenterazine (CLZN) and chemiluminescence resonance energy transfer (CRET) to the GFP moiety occurs, with an emission maximum in the green (λ = 510 nm). GA has a low Ca^2+^ binding affinity, large dynamic range of light emission, is stable and has little, if any, toxicity, making it a potentially useful reporter for application in BLI studies [13], [14].

Here, we report transgenic mice expressing a subcellularly targeted GA construct that allows non-invasive whole animal imaging of [Ca^2+^]_m_. Monitoring [Ca^2+^]_m_ can provide precise information about the role of Ca^2+ ^signaling in biological processes, such as apoptosis and the metabolic regulation of cellular respiration [15], [16]. We demonstrate that Ca^2+^-induced light emission of GA from this compartment can be non-invasively monitored with high sensitivity and over a wide temporal range from 40 milliseconds to hours. Whole body optical imaging of newborn mice identified variations in [Ca^2+^]_m_ that correlate to the ontogeny of sleep/wake cycles and motor coordination. The method offers large imaging fields of view, while details about the regulation of [Ca^2+^] in subcellular compartments can be inferred from the genetic targeting. This non-invasive approach should therefore give new insight about Ca^2+^ signaling in developmental and behavioral studies, and in mitochondrial disorders linked to muscle and nervous diseases.

## Results

### Genetic targeting for analysis of local Ca^2+^ signals

**Figure 1:**
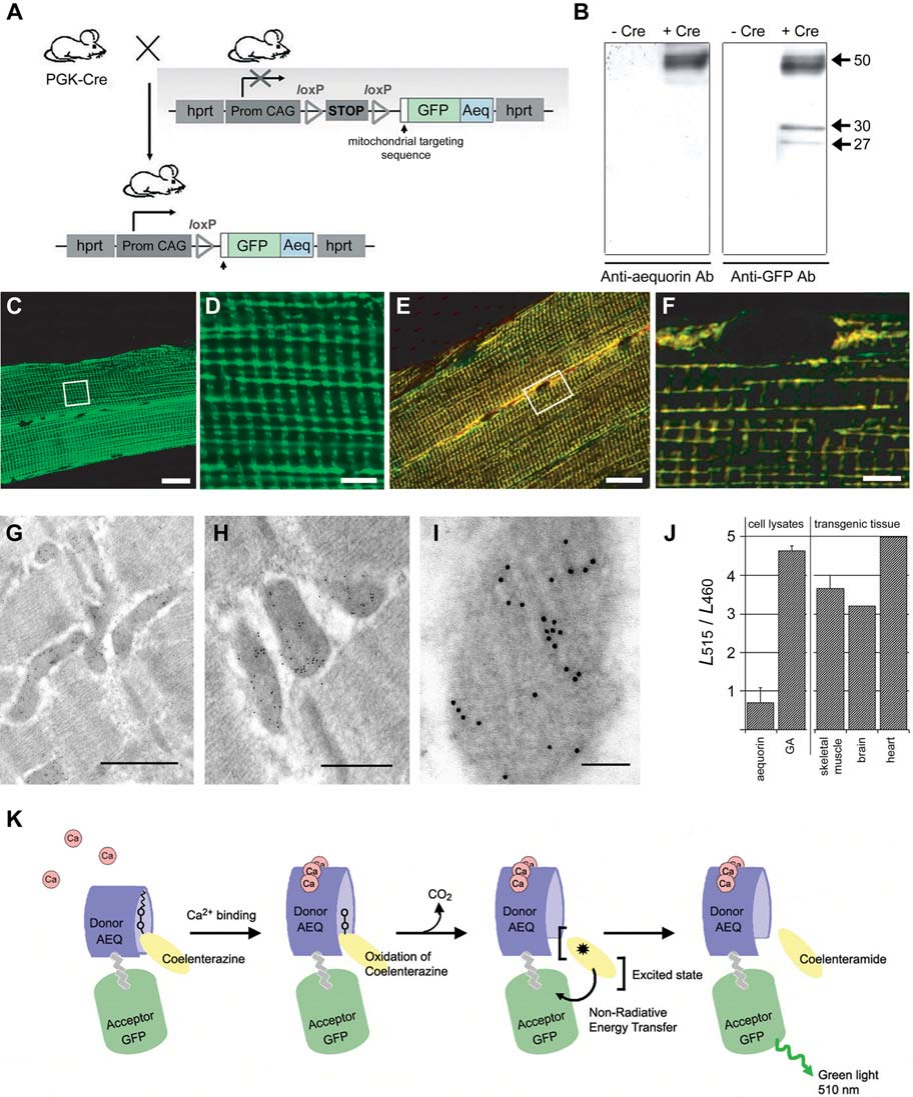
Transgenic mice constructed with Cre-inducible, mitochondrially targeted GFP-aequorin. (A) Schematic diagram showing the genetic design of transgenic animals. The transgene, pCAG-*loxP*-Stop-*loxP*-mtGA-PolyA can be activated by crossing mice with another mouse line expressing Cre under the control of different promoters for conditional activation of mtGA transcription. (B) Western blot on purified mitochondrial enriched fractions from skeletal muscle of transgenic mice crossed with PGK-Cre. Mitochondrial fractions were compared with transgenic mice, which were not expressing the mtGA protein and blots were developed with both aequorin and GFP antibodies. (C, D, E, F) Confocal analysis of mtGA fluorescence in anterior tibialis muscle fibers. (C and D) Direct fluorescence of GFP expression. (D) Enlargement of the frame area in C. (E and F) Overlay of anti-GFP labeling (green) and anti-cytochrome-C labeling (red), where yellow indicates co-localization of the two labels. (F) Enlargement of the frame area in E. Scale bars for C & E = 20 µm. Scale bars for D & F = 5 µm. (G–I). Gallery of post-embedding GFP immunogold electron micrographs from anterior tibialis mouse muscle. GFP is localized in mitochondria. GFP is visualized by sequential probing with anti-GFP antibody and IgG conjugated with 10 nm colloidal gold. Scale bars: G, 1 µm; H, 500 nm; I, 100 nm. (J) Ca^2+^ CRET activities on purified mitochondrial fractions from skeletal muscle, brain, heart and cellular extracts. CRET measurements are expressed as the ratio of green (515 nm) over blue (460 nm). (K) Schematic diagram of the GA-CLZN light reaction. The binding of Ca^2+^-ions to aequorin leads to a conformational change, which results in the oxidation of its bound substrate chromophore, coelenterazine. Non-radiative energy transfer then occurs from the excited state chromophore to GFP, which then emits light in the green (λmax = 510 nm).

Transgenic mice were generated with a mitochondrially targeted GFP-aequorin (*mtGA*) transgene. The transcription unit was introduced by knock-in to the Hypoxanthine Phosphorylated Ribosyl Transferase (*hprt*) locus on the X chromosome [17]. *mtGA* contains the targeting sequence of subunit VIII of cytochrome c oxidase for localization in the mitochondrial matrix [18]. Expression of the reporter was made conditional by using a *l*ox stop *l*ox sequence 3′ to the strong ubiquitous promoter, CAG (Figure 1A). With a conditional Cre- *l*oxP system, transcription of the transgene can be genetically activated in specific cells at a precise moment during embryogenesis or adult life.

Transgenic mice carrying the *mtGA* transgene were crossed with a PGK-Cre mouse line, in order to activate ubiquitous expression of the transgene from early stages of embryogenesis [19]. No change in phenotype was observed in the resulting transgenic mice. Western blot analysis in enriched mitochondrial fractions from skeletal muscle reveals a band at 50 kDa corresponding to the calculated molecular weight of the hybrid protein, GFP-aequorin (Figure 1B). Fluorescence detection of GFP in whole animals and tissues *ex vivo* shows that mtGA is expressed in mitochondria of major organs with a high level of expression (data not shown). Direct visualization of GFP fluorescence in fresh or fixed tissue shows expression of the reporter protein in muscle fibers of the tibialis anterior muscle (Figure 1C–1F). Electron micrographs of immunogold cytochemistry performed on skeletal muscle confirms that GFP immunoreactivity is found inside mitochondria (92.3±2.56 %, *n* = 52) (Figure 1G–1I). Finally, the ratio of light intensity at the emission maximum of the acceptor, the GFP moiety (510 nm), to that of the donor, the aequorin moiety (470 nm), was determined in enriched mitochondrial populations obtained from the skeletal muscle, heart and brain of transgenic animals (Figure 1J). The results clearly show that the optical signals originate from GFP, and that aequorin and GFP are in close enough proximity to allow transfer of the excited-state energy by CRET [13], [20] (Figure 1K), thus confirming the presence and localization of the hybrid protein in tissue from transgenic animals.

### Ca^2+^ signaling in neonatal neural tissues

**Figure 2:**
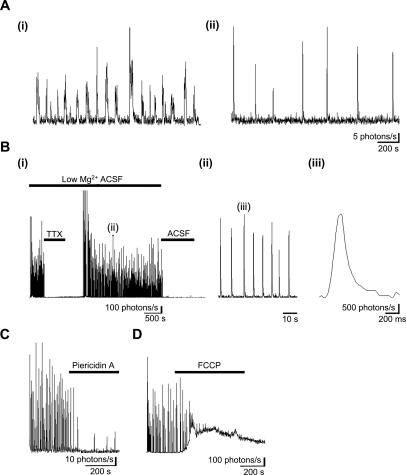
Large-scale mitochondrial Ca^2+^ signaling oscillations in acute brain slices from neonates. (A) Acute brain slices were prepared from newborn mice and prolonged recordings of bioluminescence activity was detected by microscopy in large scale areas (600 µm^2^) of the cortex. Traces represent 1500 s of data (1 s integration) obtained in the (i) temporal cortex of a horizontal brain slice from a 2 day old mouse and (ii) somatosensory cortex of a coronal slice from a 1 day old mouse. (B) Epileptiform activity induced by low Mg^2+^ in an acute coronal slice from a 10 day old mouse brain. Recording was undertaken in the somatosensory cortex. (B (i)) Low Mg^2+^ induced oscillations are blocked completely by TTX (500 nM, *n* = 3) and are absent in the presence of normal ACSF containing 1.3 mM MgCl_2_. Data in the trace is plotted with 1 s integrals. (B (ii) & (iii)) Expanded time scales at the times indicated in B (i) & B (ii), respectively. Data is plotted with 50 ms integrals. Low Mg^2+^-induced mitochondrial Ca^2+^-transients are blocked by (C) piericidin A (2 µM) and (D) FCCP (2 µM).

Two-photon imaging of acute brain slices from newborn rats previously revealed large-scale, highly synchronized early network Ca^2+^ oscillation waves (ENOs) in the developing neocortex [21]. ENOs were reported in the cytosolic compartment. In other experimental systems, Ca^2+ ^oscillations in the cytosol have been reported to occur in synchrony with [Ca^2+^] changes in the mitochondrial matrix [22]–[25]. It was therefore determined if [Ca^2+^]_m_ oscillations could be detected in acute slices prepared from the neonatal mtGA mouse brain. For the Ca^2+^ dependent CRET reaction to occur, the genetically expressed mtGA must be reconstituted with the aequorin substrate, CLZN (mtGA-CLZN). As described in previous studies [14], slices were first incubated with CLZN and then observed with a highly sensitive bioluminescence microscopy system. Using this approach, oscillations in [Ca^2+^]_m_ were detected in both the cortex and hippocampus of acute brain slices prepared from 0 to 2 day old newborns (Figure 2A (i) & (ii), *n* = 3).

These Ca^2+^ events decreased in frequency or were absent in brain slices from older animals (P4–P12) (data not shown). However, highly synchronized large-scale oscillations could be induced in brain slices from P4–P12 mice by lowering the external concentration of Mg^2+^ (Figure 2B (i)–(iii), *n* = 25) or by addition of bicuculline (10 µM) (data not shown). Blockade of Na^+^-channels with tetrodotoxin (TTX, 0.5 µM) (Figure 2B (i), *n* = 3), completely abolished the [Ca^2+^]_m_ oscillations, suggesting that they depend on neuronal activity. In addition, oscillations induced by low Mg^2+^ were reversibly blocked by the NMDA receptor antagonist, D-APV (50 µM), while those induced by bicuculline were reduced by both D-APV and the antagonist for AMPA receptors, CNQX (2 µM) (data not shown). Oscillatory rises in Ca^2+^ were absent or significantly reduced after incubation with the NADH-ubiquinone oxidoreductase (complex I) inhibitor, piericidin A (2 µM) (Figure 2C, *n* = 2) or FCCP (2 µM) (Figure 2D, *n* = 3), confirming the mitochondrial origin of these responses.

### Validation of BLI as an imaging modality for *in vivo* detection of Ca^2+^ transients during muscle contraction

**Figure 3:**
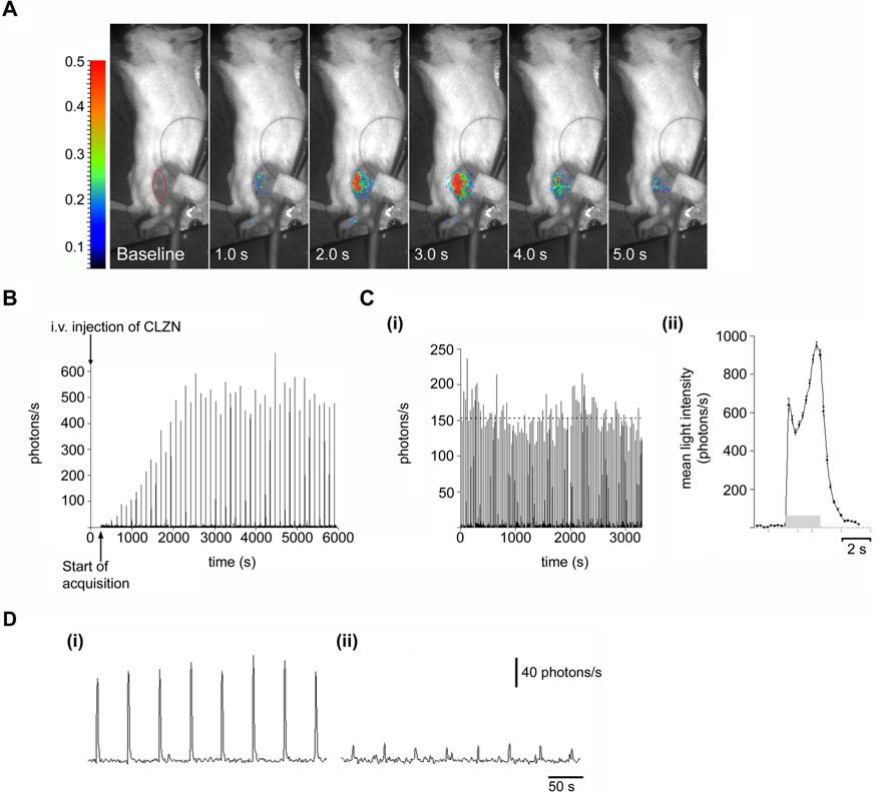
*In vivo* visualization of mitochondrial Ca^2+^ uptake in the hindlimb muscle after stimulation of the sciatic nerve. (A) Direct visualization of Ca^2+^-induced bioluminescence in the hindlimb muscle after tetanic stimulation of the sciatic nerve (5 ms pulses at 50 Hz for 2.5 s). Each frame represents 1 s of light accumulation superimposed with the video image taken before the acquisition of bioluminescence. The FOV was 16×12 cm. Smoothing has been applied to the bioluminescent image overlay to a resolution of 1 mm. Color scale is in photons/pixel/s. (B) Mice were injected (i.v) with native coelenterazine (4 mg/kg) and Ca^2+^-induced light emission was recorded immediately after in the hindlimb muscles in response to tetanic stimulations applied every 2 minutes over more than 1.5 hours. The plot shows the increasing amplitude of the light response (photons/s) as a function of time. (C (i) & (ii)) Ca^2+^-induced light emission was investigated in mice previously injected with CLZN by tail-vein and then stimulated (as described above) every 30 s for up to 1 hour, (i) Graph showing the light emission (photons/s) during repetitive nerve stimulations and contraction/relaxation cycles of the hind-limb muscle in a single mouse, (ii) The average light emission (photons/s) of the Ca^2+^ transients shown in Figure C (i) (*n = *104). The grey horizontal bar shows the time of the stimulus. (D (i) & (ii)) Trains of pulses (0.4 ms pulse duration at 70 Hz, train duration = 600 ms) were applied every 30 seconds to the sciatic nerve. Total light intensity (photons/s) was plotted over time. The graphs show the amplitude of the response (i) before, and (ii) after an intramuscular injection of Ru360 (500 µM).

Skeletal muscle contraction is initiated by the release of Ca^2+^ from the sarcoplasmic reticulum (SR), which is rapidly sequestered by mitochondria [8], [26]. In subsequent studies, we investigated if Ca^2+^-responses associated with contraction of the hindlimb muscles could be detected within intact tissues of adult mice. In these studies, CLZN was introduced via a tail-vein injection of the substrate. We found that tetanic stimulation of the sciatic nerve in mice bearing mtGA-CLZN, lead to a rapid increase in light emission in the hindlimb muscles (5 ms pulses, 50 Hz, 2.5 s train duration) (Figure 3A and Movie S1). In contrast, no increase in light was ever detected in wild-type mice that had been injected (i.v.) with CLZN and subjected to the same stimulation protocols (data not shown).

Other studies reporting whole animal bioluminescence imaging of gene expression with *Renilla* luciferase, indicate that CLZN has limited access to some tissues and that it can be unstable in biological media [27]–[29]. The time course for formation of the mtGA-CLZN complex was examined by monitoring the muscle signal triggered by nerve stimulation at repeated time intervals after tail-vein (i.v.) injection of CLZN in mice. The time required for the CRET signal to reach maximum amplitude following CLZN injection ranged between 15 and 35 min (*n* = 4 mice, see Figure 3B for an example). The light responses showed a high degree of reproducibility after repetitive tetanic stimulations and contraction/relaxation cycles in the hindlimb muscles. Remarkably, [Ca^2+^]_m_ transients corresponding to tetanic contraction of the hindlimb muscle could be detected for up to 100 stimulations over a 1–2 hour period (Figure 3C (i)), and their amplitude and kinetic profiles were essentially constant (Figure 3C (ii), *n* = 104). Similar optical signals were recorded when the same mouse was re-injected with CLZN on different days. Intramuscular injection of Ru360, a specific inhibitor of mitochondrial Ca^2+^ uptake, attenuated Ca^2+^ rises evoked by tetanic stimulation (70 Hz) of the sciatic nerve (Figure 3D (i) & (ii)).

**Figure 4:**
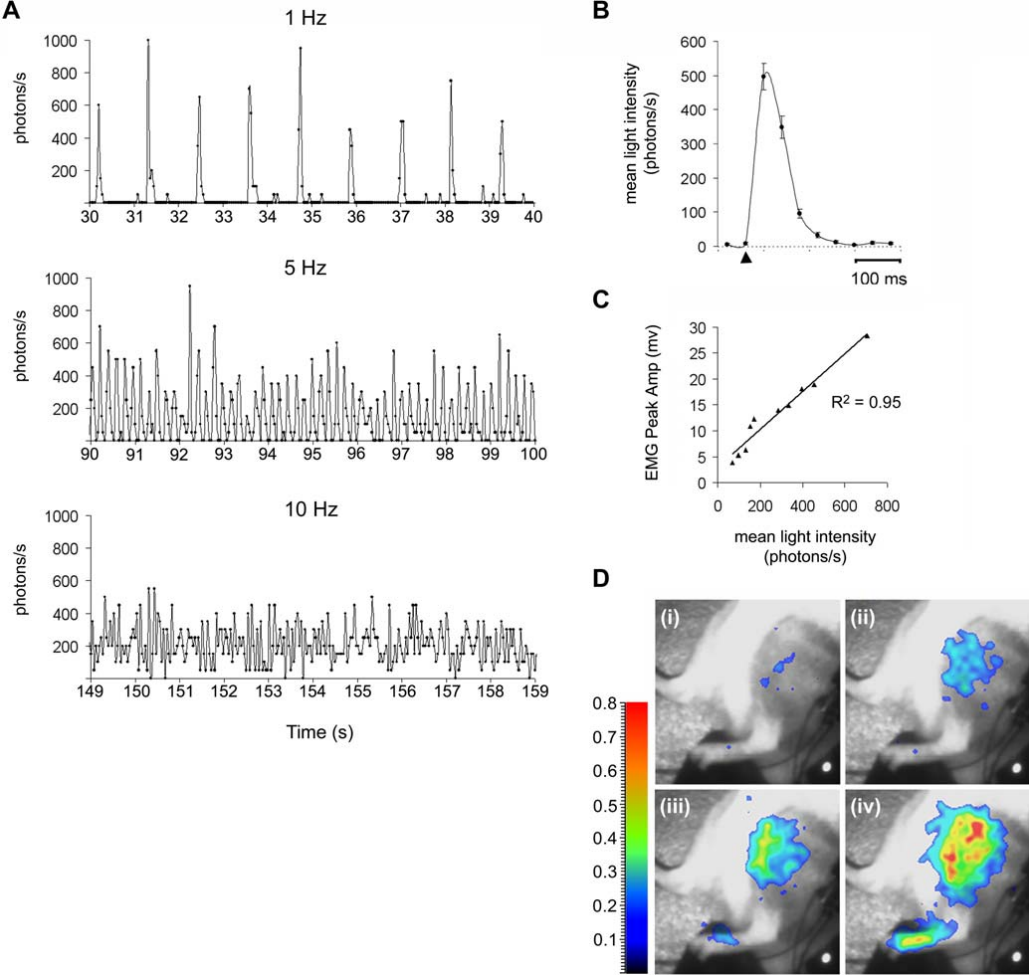
*In vivo* detection of Ca^2+^ transients during single twitch and tetanic contractions of the muscle. Trains of stimuli at different frequencies were applied to the sciatic nerve and mitochondrial Ca^2+^ uptake was monitored in the hindlimb muscle using an acquisition rate of 25 Hz. (A) Graphs showing light emission detected from the muscle after trains of stimuli (10 s duration) were applied to the sciatic nerve at 1, 5 and 10 Hz. The data at each time point on the graph represents 40 ms integrals. (B) The average light emission of Ca^2+^-transients plotted with 40 ms integration during single-twitch muscle contractions (1 Hz stimulation) (*n* = 35, 4 mice). Error bars show±s.e.m. (C) Plot of the variation of the peak amplitude electromyogram *versus* the maximum light intensity when varying the stimulation voltage. (D) Bioluminescence superimposed with the video image of a transgenic mouse hindlimb after stimulation of the sciatic nerve at (i) 1 Hz, (ii) 5 Hz, (iii) 10 Hz and (iv) 50 Hz. The FOV was 16×12 cm. Smoothing has been applied to the bioluminescent image overlay to a resolution of 1 mm. Color scale is in photons/pixel/s.

[Ca^2+^]_m_ is known to be involved in the regulation of oxidative phosphorylation and apoptosis, but its role in skeletal muscle function is still largely unknown [16]. A recent study by Rudolf et al. characterized changes in [Ca^2+^]_m_ during contraction of skeletal muscle fibers expressing the Ca^2+^-sensitive fluorescent protein, yellow cameleon (YC2) [8]. They used two-photon microscopy to record cytosolic and mitochondrial Ca^2+^-responses with good spatial resolution in intact tibialis anterior muscle fibers, *in situ*. Applying the same protocols for stimulation of the sciatic nerve as Rudolf et al., we obtained comparable results with BLI. However, in our studies we monitored light responses from the entire hindlimb muscle (or at least a large number of fibers from that muscle) using a larger field of view and a lower spatial resolution. 10 s trains of stimuli were applied every 60 s to the sciatic nerve at different frequencies (1–50 Hz) (Figure 4A–4D, *n* = 4 mice). Results show that mitochondrial Ca^2+^ uptake/release during single twitch muscle contractions (1 Hz) can be recorded *in vivo*, with a time resolution down to 40 ms (Figure 4A). The recorded light responses were well correlated to different frequencies of sciatic nerve stimulation (1–10 Hz). Furthermore, when the mean light intensity was plotted after applying a train of stimuli at 1 Hz, the profiles of the corresponding Ca^2+^-transients were highly reproducible (Figure 4B, *n = *35, 4 mice). The intensity of the muscle contraction, i.e. the number of muscle fibres recruited, also increased with an increased stimulation voltage and there was a strong correlation between the mean light intensity and the peak amplitude of the electromyogram (Figure 4C). This is in line with *in vitro* studies, which suggest that mitochondrial Ca^2+^ uptake is quantitatively correlated to the force of the muscular contraction [30]. Differences in the intensities of light emission were also correlated to the frequency of sciatic nerve stimulation (Figure 4D (i)–(iv)). Overall, our data for muscle contraction confirm the results of previous studies, validating this completely non-invasive method as suitable for functional imaging of Ca^2+^ responses during studies on muscle contraction.

### Periodic variations in mitochondrial [Ca^2+^] in whole body imaging of newborn mice

**Figure 5:**
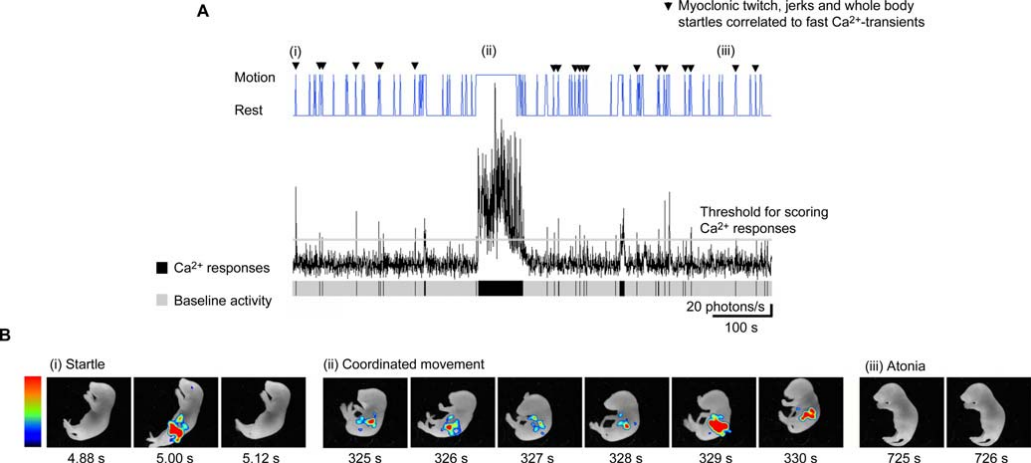
Distinct mitochondrial Ca^2+^ responses in newborn mice are linked to different behavioral states. A newborn mouse (P1) was injected (i.p.) with coelenterazine and whole animal bioluminescence was co-registered with the video image with an acquisition rate of 25 Hz using the Video Imager. (A) Graph shown in black is the total light intensity (photons/s) detected during more than 700 s of recording. The thin grey line running through the graph shows the minimum threshold set for scoring Ca^2+^-responses whose amplitudes reach or go above this limit. The upper blue graph shows the cycling between motion and rest, which was determined by visual analysis of the video frames. Closed black arrow heads show where fast Ca^2+^-transients (<1 s duration) are correlated to muscular twitches or whole body startles. Sustained Ca^2+^-responses (>1 s duration) are associated with coordinated movements. The lower grey bar shows periods of baseline Ca^2+^ activity (below the threshold line set) together with black bars that represent the Ca^2+^-responses and their durations. (B) Examples of the three behavioral states visually characterized; including (i) whole body startle, (ii) co-ordinated movement and (iii) atonia. Data for each state represents the video image superimposed with the bioluminescence image at the times indicated on each frame and in Figure 5A. (i). Frames represent 40 ms integration of the bioluminescence superimposed to the corresponding video image. Color scale is 0.000725–0.00145 photons/pixel (5 mm smoothing has been applied). (ii & iii) represents 160 ms integration of the bioluminescence superimposed to the corresponding video frame. Color scale is 0.00174–0.00377 photons/pixel (3.5 mm smoothing). The FOV used in these studies was 8×6 cm.

In subsequent studies, [Ca^2+^]_m_ responses associated with movement were recorded in non-anaesthetised and unrestrained freely moving CLZN-injected newborn mice (Figure 5A, (*n* = 6)) using an imaging system that allows co-registration of the video and bioluminescent images. Three major behavioral states, corresponding to the known components of sleep/wake cycles, were identified: (i) whole body startles and myoclonic twitches (see Movie S2 for an example), (ii) coordinated movements (see Movie S3 for an example) and (iii) atonia (Figure 5B). In sleep/wake cycles of newborn rats or mice, myoclonic twitching is the most reliable way to identify the presence of active sleep [31], [32]. In line with the data from contraction/relaxation cycles of the hindlimb muscle, whole body startles or muscular twitches identified in the video recordings were correlated to fast Ca^2+^-transients having short durations (<1 s) (Figure 5A & 5B (i)). In contrast, coordinated movements were made up of sustained Ca^2+^ rises occurring across large scale areas of the body (Figure 5A & B (ii)).

**Figure 6:**
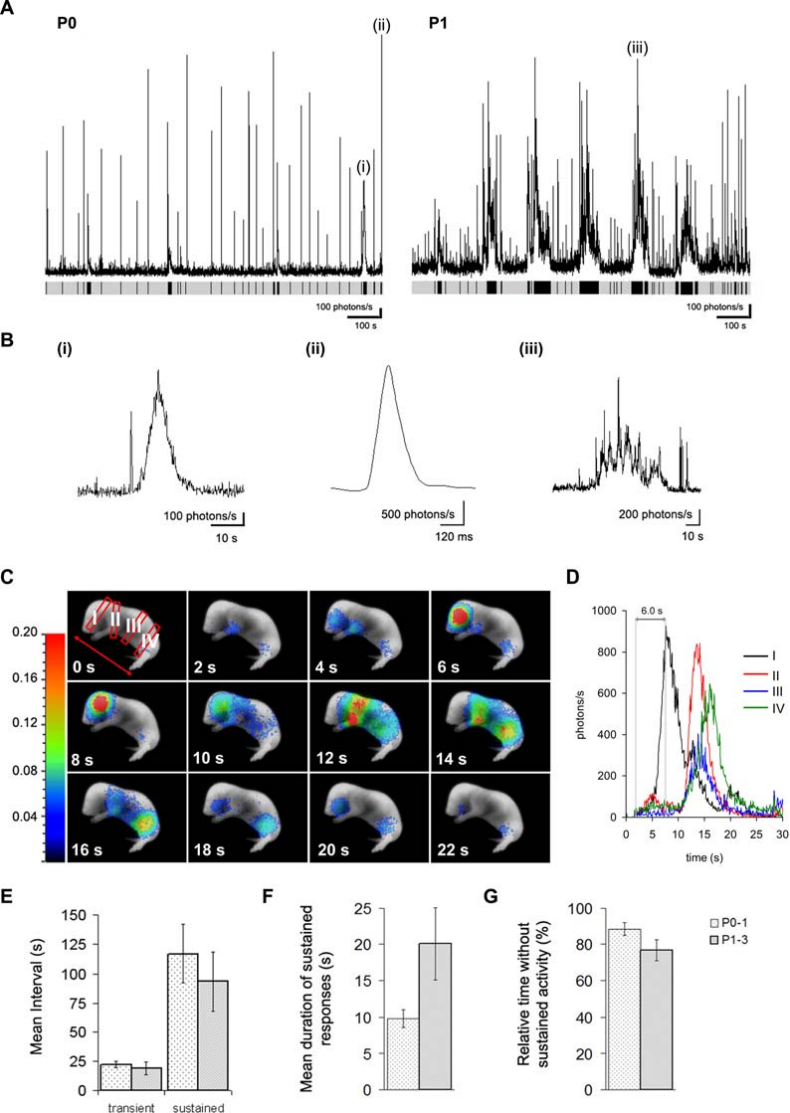
Variations in mitochondrial [Ca^2+^] are related to behavior in newborn mice. Newborn mice expressing mtGA were injected (i.p.) with native coelenterazine and bioluminescence activity was recorded in un-restrained and freely moving newborns. (A) Recording traces showing whole body Ca^2+^-transients in newborn animals over 1000 s. (P0) Newborn mouse (<12 hours old, P0) and (P1) a 1 to 2 day old mouse pup. The grey bar below plots shows periods of baseline Ca^2+^ activity together with the Ca^2+^-responses in black (as for Figure 5). (B (i), (ii) and (iii)), expanded time base of the different Ca^2+^ responses indicated in (A), including sustained Ca^2+^ responses (i) & (iii) and Ca^2+^ transients of short durations (ii). (C) Sequence of consecutive images (video image superimposed with the bioluminescence image). A video image was taken at the beginning and at the end of a short recording period during which a sustained and coordinated increase in Ca^2+^-induced light emission was detected across the entire body. Each frame represents 2 s of light accumulation. Smoothing has been applied to the bioluminescent image overlay to a resolution of 1 mm. Color scale is in photons/pixel. (D) Corresponding graph showing the time course of the Ca^2+^-responses in regions of interest shown in the first frame in Figure 6C. (E–G) Histograms comparing data from animals less than 1 day old (P0–1; *n* = 9; dotted columns) and animals between 1 and 3 days old (P1–3; *n* = 8; light grey columns). (E) Histogram showing the mean time interval between fast Ca^2+^-transients and sustained Ca^2+^ responses. (F) Histogram showing the mean duration of sustained responses. (G) Histogram showing the total percentage of time in the absence of sustained activity. The FOV used in these studies was 8×6 cm.

In further studies, bioluminescence was monitored in newborn animals over longer recording durations (1–1.5 hours). Similarly to studies on the hindlimb muscle, signal was detectable within 15 minutes, and for up to 10 hours after i.p. injection in pups that had been returned to their litter (*n* = 6). Variations in [Ca^2+^]_m_ detected from whole body recordings of newborn mice had distinct patterns (Figure 6A). Whole body Ca^2+^-response patterns were observed to be made up of two major forms (a) fast Ca^2+^ transients having short durations of 100's of ms to 1 s, which were predominant (Figure 6 A(ii)) and (b) sustained Ca^2+^ responses (>1 s) (Figure 6A (i) & (iii)). Based on the observed patterns, we separated newborn animals into two groups, one group of P0–1 (*n* = 9) and another group of P1–3 (*n* = 8). Sustained Ca^2+^ responses had highly variable durations in both groups (>1–200 s) (Figure 6B (i) & (iii)). In addition, fast Ca^2+^ transients were often observed to precede sustained responses (see Figure 6B (i) for example). In some cases, sustained responses were observed to occur across the entire body in a coordinated fashion (Figure 6 C & D, *n* = 4). The mean interval between fast Ca^2+^ transients and sustained responses was not significantly different between the two groups (Figure 6E). However, animals from the group at P0–P1, had sustained responses with durations that were approximately half as long (9.8±1.2 s, *n* = 9 mice, 149 events) compared to older P1–P3 animals (20.2±5.0 s, *n* = 8 mice, 151 events) (Figure 6F). Sustained Ca^2+^ responses in the older animals also had more complex patterns (see Figure 6B (i) *versus* 6B (iii)). Overall, the percentage of time recorded in the absence of sustained Ca^2+^ responses was 88.5±3.5 % (*n* = 9) and 76.9±3.5 % (*n* = 8) for P0–1 and P1–3, respectively (Figure 6G). Recent studies using fiber optics implanted in the cortex of non-anaesthetised newborn mice showed that spontaneous and synchronized ENOs occur in the cortex during intermittent sleep-like periods, which are absent during motion [9]. In addition, *in vitro* studies on acute brain slices confirm that spontaneous and experimentally induced Ca^2+^ transients are readily detectable in neural tissues from the mtGA mouse (Figure 2). However, we did not detect CRET signals from the brain of newborn mice administered with CLZN, neither by i.p. injection (*n* = 3), nor when the substrate was directly injected into the brain (*n* = 3).

### *In vivo* detection of bi-laterally synchronized Ca^2+^ responses during seizures

**Figure 7:**
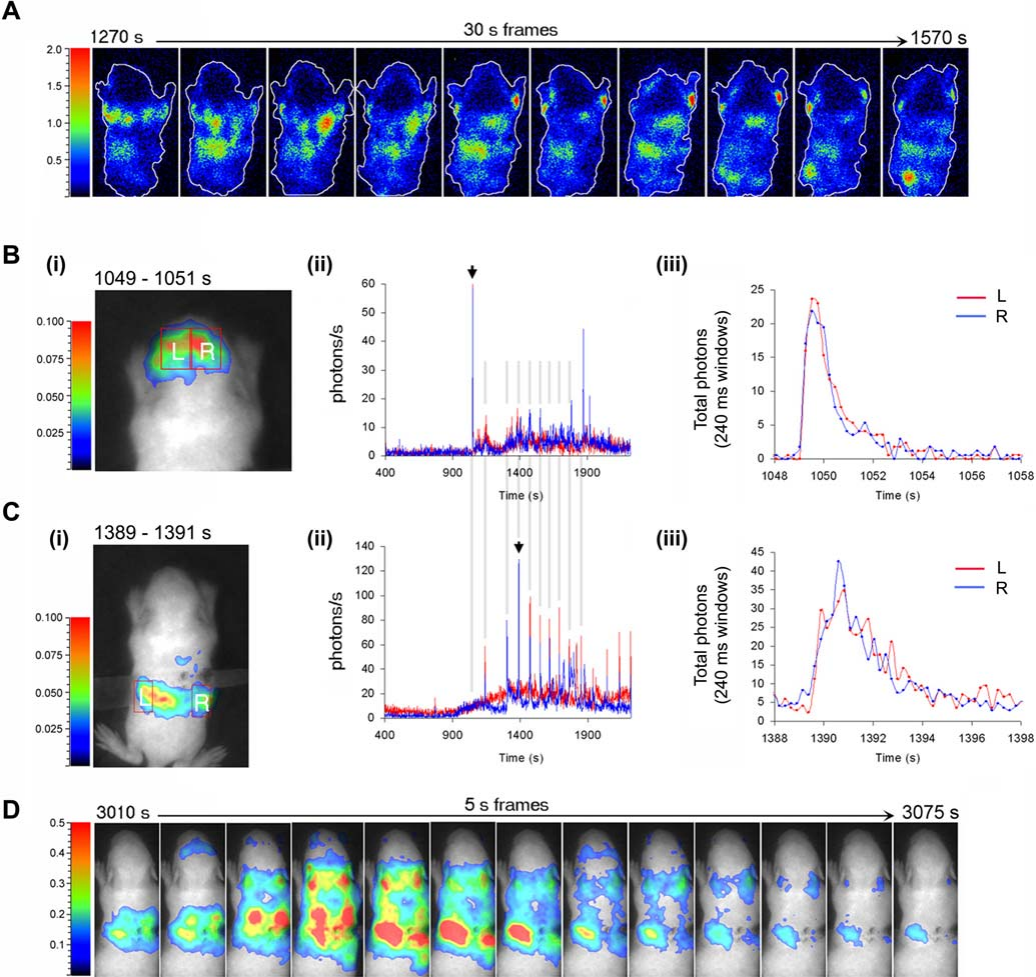
Visualization of mitochondrial Ca^2+^-fluxes during kainic acid-induced seizure.

We next investigated if Ca^2+^-transients could be detected during kainate-induced seizure in non-anaesthetised 6–7 day old mice. Imaging was started immediately after i.p. injection of kainic acid (25 mg/Kg) and was continued for up to 1 hour. In the sequence of images shown (Figure 7A and Movie S4), the dynamic patterns of Ca^2+^ distribution recorded reveal a remarkable profile of the mouse undergoing a seizure, characterized by clonic movements of the forelimbs [33]. The recorded photon flux in selected regions of interest plotted as a function of time indicates that a marked increase in [Ca^2+^] levels occurred in all areas approximately 15 minutes after drug administration (Figure 7B & C). Elevated Ca^2+^ activity appeared both as a slow rise in the basal concentration and as oscillations in which Ca^2+^ spikes only lasted a few seconds (Figure 7B & C, (ii) & (iii)). Based on other studies, kainate receptors are not only present in the brain, but also in the spinal cord, adrenal glands, testis and the gastroenteropancreatic system [34], [35]. Analysis of several regions of interest indicated that highly synchronized, bi-lateral Ca^2+^ transients also occurred in a spatially localized region on the dorsal side of this animal (see Figure 7C (i)–(iii)). These Ca^2+^ transients were faster than those accompanying coordinated muscle movements and they may therefore be linked to adrenal activity, which is expected to be high during states of stress. Some of the signals were also synchronized along the dorso-ventral axis. Hence, signal transduction can be monitored along the rostro-caudal, dorso-ventral and lateral axes in whole living animal models of epilepsy. Finally, using bioluminescence for imaging over long periods can also reveal other interesting phenomena, such as Ca^2+^ waves occurring across the entire animal, which were observed after kainate induced seizures (Figure 7D (*n* = 5) and Movie S5).

## Discussion

A challenge in biology is to monitor signal transduction in a *bona fide* physiological context, i.e. in live, un-restrained and non-anaesthetised animals. Our objective here was to develop an approach allowing non-invasive *in vivo* detection of local Ca^2+^-signaling in freely moving animals. A genetic approach was chosen because it allows specific *in vivo* targeting, endowing optical signals with a precise knowledge of their origin even when detection takes place from outside of the living animal. In previous studies we showed that the Ca^2+^-sensitive genetically encoded bioluminescent protein GFP-aequorin, could be expressed in several organelles such as the mitochondrial matrix for detection of localized [Ca^2+^] in cells [14]. Here, we demonstrate that GFP-aequorin, targeted to the mitochondrial matrix, can also be stably expressed in transgenic animals at all stages of development. Monitoring mitochondrial [Ca^2+^] will enable the role of this compartment to be examined in physiological processes (e.g. biological rhythms, learning & memory, ageing, energy metabolism & apoptosis) and in pathological conditions (e.g. excitotoxicity and metabolic disorders).

When transgenically expressed mtGA is reconstituted with coelenterazine (mtGA-CLZN), it provides a high signal over background, allowing elevations in the Ca^2+^ concentration of the mitochondrial matrix to be non-invasively investigated. Whole animal recordings of light emission during induced Ca^2+^ concentration changes in hindlimb muscle mitochondria during contraction/relaxation cycles are well correlated with previously reported data obtained by fluorescence microscopy in both *in vivo* [8] and *in vitro* [8], [26] studies. Up to now, *in vivo* imaging of calcium signaling has remained more or less invasive, precluding its use in freely moving, unrestrained and behaving animals. In contrast to methods using fluorescence imaging, bioluminescence does not require light excitation and the high contrast-to-noise ratio afforded by mtGA-CLZN is based on the absence of background bioluminescence in tissues. Furthermore, the acquisition of signals is undertaken with a photon counting system based on a cooled GaAs intensified charge-coupled device having no readout noise. The importance of this approach is that the imaging parameters (e.g. spatial binning and signal integration) do not need to be predefined. This opens up the possibility to detect Ca^2+^ signals covering a broad temporal range (from 100's milliseconds to 100's seconds), which can be identified in post-processing of the data according to signal intensities and kinetic properties.

The main advantage of imaging CRET with mtGA-CLZN lies in its total absence of invasiveness. This new method is therefore extremely well adapted to the analysis of calcium signaling in behavioural states. Its trade-off is a limited spatio-temporal resolution due to the low fluxes of collected light. In theory, the intrinsic characteristics of the detection system used here would allow a temporal resolution as low as 40 ms (acquisition rate at 25 Hz) and in the absence of light absorption and scattering by tissues, a spatial resolution of 100 µm or 200 µm depending on the field of view chosen by the operator (8 by 6 cm or 16 by 12 cm, respectively). Schematically, the photon fluxes reaching the detection system are a combination of (i) the photon emission rate, which depends on the biological construction and the biological activity, and (ii) the absorption and scattering of photons by the living tissues, which depend on their depth of emission and their wavelength. This latter component cannot be easily measured and is unaccounted for in the estimation of the “true” spatial resolution of the images. In practice, either the temporal or the spatial resolution, or both, need to be decreased in order to allow sufficient statistics of photon counting. In the different experiments reported here, which represent a panel of different levels of light emission in different volumes of biological tissue, either the intrinsic temporal resolution limit (40 ms) was reached at the extent of a relatively large degradation (i.e. smoothing) of the spatial resolution [example in Figure 4D, 1 mm smoothing in a muscle contraction experiment]; or alternatively, the temporal resolution was largely degraded (i.e. integration of sequential time frames) in order to achieve superior spatial resolution [example shown in Figure 7A, 30 s integration times for a spatial resolution of 300 µm]. In any case, these numbers do not take into account the non-linear relationship between the signal intensity and the degree of light scattering and absorption in tissues, for which no formal solution can be computed. In addition, the trade-off between spatial and temporal resolution is a classic problem of all imaging techniques with low count numbers, such as for instance Positron Emission Tomography (PET). Similarly to PET, the software used here allows list mode acquisition of events and *a posteriori* reconstruction of the image set in one or several optimized sequence combinations, without loss of quantitation accuracy. In particular, the smoothing algorithms use unitary gain filters that have no effect on output values as long as measurements are made in a region of interest larger than the smoothing kernel (see Methods).

In its present state of development, the mtGA-CLZN/CRET method offers spatial resolution at a tissular level in whole mice combined with sub-second temporal resolution, and its advantage over invasive but more resolutive techniques must be weighted on a case-by-case basis. However, several lines of technical development suggest that improvement of both spatial and temporal resolutions are feasible. Firstly, it appears possible to decrease the flux of photon absorbed in tissue by GA-like constructs with red-shifted photoproteins [36]. Second, coupling of the GA elements could be optimized for better resonance energy transfer. Third, the efficiency of light collection by the detection system could be improved by clever geometries. Future developments will tell us if cellular resolution can be achieved with this approach.

Transgenically expressed GA-CLZN was found to be remarkably stable and Ca^2+^ signals could be monitored over hours, which is ideal for undertaking physiological measurements in behavioural studies. The organisation of early behaviours can be categorised on the basis of their spontaneity and coordination. Co-registration of whole body bioluminescence imaging of Ca^2+^ signaling with video records of behaviour, identified that fast Ca^2+^-transients and sustained Ca^2+^-responses were well correlated with spontaneous muscular twitches and coordinated movements, respectively. These results indicate that whole body imaging of Ca^2+^-signaling can be used as a molecular imaging technique to identify three major behavioural states in newborn mice, namely atonia, muscle twitching or startles and coordinated movements [32], [37], [38]. These behavioural states are among the criteria used to define sleep in neonates at ages when EEG is not a reliable marker [39], [40]. In infant rats or mice, investigators typically rely on measures of body movements or the nuchal muscle tone in order to categorize sleep. In general, periods of wakefulness entailing voluntary and coordinated limb movements are followed by periods of sleep characterised by atonia and myoclonic twitching of the limbs, before another period of wakefulness ensues. This rhythmic activity otherwise known as the sleep/wake cycle, undergoes rapid cycling in newborn animals [9], [38], [39]. Indeed, the patterns of Ca^2+^-responses obtained from whole body recordings had characteristics analogous to these sleep/wake cycles. Overall, our video records of behaviour indicated that motor patterns in newborn mice were dominated by spontaneous muscle twitches, limb jerks and whole body startles [41]. Similarly, the total time between sustained Ca^2+^-responses (i.e. when fast Ca^2+^ transients were frequent), was calculated to be close to 80 % for newborn animals. This is similar to the value given for the total time spent by newborns in sleep states [9], [38], [42]. The duration of sustained Ca^2+^ responses recorded in our studies was also compatible with data reported for periods of wakefulness in infant mice and rats [38], [39].

Myoclonic twitching is believed to play a critical role in early motor and somatosensory development. In particular, temporal correlation among motor and sensory events is believed to modify synapses through a Hebbian type learning process [43]. Indeed, sensory input from early muscular activity has been found to precede bursts of network activity in the developing somato-sensory cortex of newborn rats [44]. Buzsaki and co-workers suggest that the influence of physiological sensory input from spontaneous uncoordinated and coordinated skeletal movement allows the three dimensional shape and mechanical properties of the body to be established by the sensorimotor system, which would in turn allow sensorimotor coordination to develop. It will be interesting to investigate whether the duration of sustained Ca^2+^ responses (i.e. coordinated movements) are linked to ontogenic adaptation in the mtGA mouse.

The relationship between Ca^2+^ responses in the neocortex and motion episodes was recently studied in newborn mice [9]. Synchronized Ca^2+^ oscillation waves, called early network oscillations, intrinsic to the neocortex, occur on average every 25 s during intermittent sleep-like resting periods. Early network oscillations could not be examined in our studies due to the difficulty of detecting brain activity in mice expressing the mtGA probe. However, spontaneously driven Ca^2+^ transients in acute brain slices from transgenic mtGA newborn mice were readily detectable. It will be necessary in future studies to confirm if Ca^2+^ oscillations in the mitochondrial matrix parallel the intracellular Ca^2+^ concentration changes associated with early network oscillations, which could be important at least in the context of balancing energy demands during critical stages of development.

The major advance of this approach is that it enables optical detection of localized Ca^2+^ signals in un-restrained and non-anaesthetised animals. Naturally, this new technique bears some limitations, such as the difficulty to detect Ca^2+^-signals in deep tissues like the brain. The present construct does not allow Ca^2+^ peaks to be detected from the brain *in vivo* for two reasons. Firstly, light with shorter wavelengths (e.g. blue & green) is strongly absorbed by tissue [45], therefore transmission of mtGA light signals through the skin and the skull is largely attenuated. Secondly, coelenterazine is a substrate for *p*-glycoproteins that are highly expressed at the blood brain barrier, restricting coelenterazine access to the brain [29]. At present, transgenic mice expressing GFP-aequorin constructs are useful for studies of peripheral organs and in particular of muscular function. In the future, new probes under development based on red-shifted variants of GFP-aequorin may improve the sensitivity to undertake imaging of the brain and other deep tissues, like the liver and the heart [36].

Changes in the position or the angle of freely moving mice or body parts in relation to the CCD camera may also modify the amplitude and kinetics of the light response detected. Sophisticated algorithms for tracking regions of interest on freely moving animals together with the implementation of different approaches, such as stereoscopy, triangulation and spectroscopic methods are under development in order to solve these projection artifacts. Quantification may also be improved by co-registration of another expressed construct, emitting Ca^2+^-independent bioluminescence at a different spectral wavelength (i.e. one that contains a different fluorescent acceptor, such as *Renilla* luciferase coupled to YFP, which also uses the same co-factor).

The method can also be adapted to different applications. For example, the Ca^2+^ sensitivity of aequorin could be modified using commercially available analogs of coelenterazine [46]. Alternatively, a mutant version of apoaequorin could be used in place of the native version inside the transgene, to lower its Ca^2+^ binding affinity [47]. Cell specific promoters for Cre-recombinase could also drive expression of the transgene in selected cell types and new lines of mice could be developed where the reporter is targeted to other subcellular domains. Mice carrying GA transgenes can also be crossed with disease model mice (e.g. for neurological or muscular disorders) to follow abnormalities in Ca^2+^ homeostasis, at the cellular, tissue and/or systems level.

In addition to the applications of whole animal *in vivo* Ca^2+^-imaging highlighted above, other imaging reporters based on the resonance energy transfer (e.g. BRET, CRET) mechanism can be genetically encoded in animals and measured under similar dynamic conditions. For instance, live imaging of protein-protein interactions through the separate expression of 2 chimeric proteins, one linked to an ‘acceptor’ and the other to a ‘donor’ protein, which will emit BRET when they come in close proximity (<10 nm) [48], [49]. This will require quantitative measurements at different spectral wavelengths.

This is the first direct recording of Ca^2+^ signals *in vivo* at the whole mammalian level where large-scale spatio-temporal information can be obtained about the role of intracellular Ca^2+^ signaling in the highly coordinated activity of muscle groups in the intact animal. In addition, rapid imaging of Ca^2+^-signaling in freely moving animals is feasible and fundamental molecular mechanisms can now be explored non-invasively, opening new avenues for studies on the role of Ca^2+^ signaling in behavior or muscular function in more relevant physiological contexts.

## Methods

### Mice

Animal manipulation and husbandry were in agreement with the European Commission, directives of the French Research Ministry (885, 3180, 3181/2005 to P.B).

### Generation of transgenic mice

Transgenic mice were generated using homologous recombination in embryonic stem (ES) cells to produce transgene insertion at the Hypoxanthine Phospho Ribosyl Transferase (*hprt)* locus on the X chromosome [17]. GFP-aequorin hybrid gene (G5A) fused to the mitochondrial matrix targeting sequence of COX VIII [18] (Figure 1A), was cloned into the pENTRY vector (Invitrogen, CA). The pCMV-chicken β-actin (CAG) promoter and the *lox*P-stop-*lox*P sequence (for conditional expression of the transgene) were cloned into the pBluescript cloning vector (Stratagene, U.S.A). The pCAG-*lox*P-stop-*lox*P cassette was then subcloned to the 5′ end of the *mtGA* gene in pENTRY and a *poly A* sequence of the rabbit β-globulin was added 3′ to the *mtGA* sequence. The transcription unit was introduced by targeted insertion into ES cells by homologous recombination at the *hprt* locus. During this process, the *hprt* gene was functionally restored by human *hprt* DNA sequences. *Hprt* is a ubiquitously expressed locus, ensuring minimal variation in expression levels of the reporter in cells so that analyses can be made both at the systems and single-cell level. Genetically modified ES cells containing the *mtGA* transgene were then injected into blastocysts (Nucleis, Speedy Mouse® technology). Animals were identified by polymerase chain reaction (PCR) using a reverse primer localized inside of aequorin and a sense primer inside the Lox stop:

AeGFP.rev: TCAGTTATCTAGATCCGGTGG;

LoxSTOP S: CGGGAAAAAGTTAGTTGTGG.

The amplified 2.1 kb fragment covering most of the transgene was DNA sequenced from transgenic mouse tissues.

In these studies, activation of the *mtGA* transgene was induced by crossing with the PGK-Cre transgenic mouse strain, which has ubiquitous expression of the site-specific Cre recombinase [19]. A stable transgenic mouse line was generated that expresses the mtGA reporter in cells from early stages of embryogenesis. Transgenic mice were routinely genotyped by PCR of aequorin, Cre & GFP on tail genomic DNA.

### Gel electrophoresis and immunoblotting

Purified fractions of mitochondria were obtained at 4°C from adult transgenic mice using a similar method as previously reported [50]. Briefly, mice were euthanised by CO_2_ inhalation and tissues were quickly excised. Tissue was homogenized in the following solution [mM: 100 KCl, 40 Tris.HCl, 10 Tris base, 5 MgCl_2_, 1 EDTA, and 1 ATP, EDTA-free protease inhibitor cocktail (Complete™, Roche), pH 7.4 at 4°C]. The preparation was then centrifuged (×2), at 800×*g* for 10 min. Each time the supernatant was then collected and centrifuged again at 9,000×*g* for 10 min to pellet the mitochondrial population, which was resuspended in [mM: 100 KCl, 10 Tris.HCl, 10 Tris base, 1 MgSO_4_, 0.1 EDTA, and 0.02 ATP, and 1.5% BSA, EDTA-free protease inhibitor cocktail (Complete™, Roche), pH 7.4], and centrifuged at 8,000×g for 10 min. Further mitochondrial purification was performed by Ficoll gradient as previously described [51]. The final purified mitochondrial pellet was resuspended in [mM: 230 mannitol, 70 sucrose, 10 Tris-HCl, 1 EDTA, EDTA-free protease inhibitor cocktail (Complete™, Roche), pH 7.4].

SDS-PAGE was performed according to the method of Laemmli (1970), using 12% gels. Immunoblotting was undertaken on Immobilon-P membranes (Millipore, Bedford, MA). Incubations were undertaken first with rabbit polyclonal antibodies (anti-GFP Biovalley, anti-aequorin Abcam) in PBS containing 5 % milk supplemented with 0.3% Tween-20, and immunoreactive proteins were then observed by incubation with HRP-conjugated goat anti-rabbit IgG antibodies followed by enhanced chemiluminescence (ECL) western blotting detection reagents (Amersham, France).

### Histology and immunohistochemistry

The complete anterior tibialis muscle was removed, washed in PBS 1X solution then fixed for 1h in PFA 4% solution (in PBS, pH 7.4). After washing in PBS 1X solution, the muscle was teased apart in PBS to obtain isolated fibers. GFP fluorescence was directly visualized in fibers after washing in PBS and mounting in FluorSave reagent (Calbiochem, USA). For the immunofluorescence studies, non-specific antibody binding sites were blocked by incubating fixed muscular fibers for 30 min in PBS containing 10% normal goat serum (NGS)/0.25% Triton X-100. After incubation, the fibers were immunostained with a 1∶500 dilution of anti-GFP polyclonal antibody (Biovalley, France) and a 1∶250 dilution of anti-cytochrome C monoclonal antibody (BD Biosciences) in PBS containing 2% NGS/0.25% Triton X-100/0.2% Bovine Serum Albumin (BSA) washed with PBS containing 0.25% Triton X-100/0.2% BSA several times, incubated with a 1∶1000 dilution of a Cy™3 conjugated anti-mouse antibody (Jackson ImmunoResearch) and a 1∶1000 dilution of a Alexa®Fluor 488 goat anti-rabbit antibody (Molecular Probes, Inc.). The stained preparation was then mounted in FluorSave reagent. Confocal analysis was performed using an Axiovert 200M laser scanning confocal microscope (LSM-510 Zeiss; version 3.2) through a 63×/1.4 NA, oil-immersion objective using LP560 and BP505-550 filters. The pinhole aperture was set at 98 µm and images were digitized at 8-bit resolution into a 512×512 array.

### Immunoelectron microscopy

Anterior tibialis muscle was dissected out and fixed for 1 h with 4% PFA (Electron Microscopy Sciences, Hatfield, PA, USA) in 0.1 M phosphate buffer (PB, pH 7.4). Muscle blocks were post-fixed for 30 min with PBS containing 4% PFA, 0.1% glutaraldehyde (TAAB Laboratories, Aldermaston, UK) and 0.2% picric acid (Sigma-Aldrich), followed by 0.5% osmium tetroxide (Electron Microscopy Sciences) for 30 minutes in the same buffer and embedded in LR-White resin (Electron Microscopy Sciences). The post-embedding inmunogold GFP labeling was followed as previously reported [52]. Thin sections were incubated with a polyclonal rabbit anti-GFP antibody (MBL, 1∶40 dilution) for 90 min followed by 60 min with goat anti-rabbit IgG conjugated with 10 nm gold (1∶25 dilution). After gold fixation with 2.5% glutaraldehyde, samples were counter stained and observed in a Jeol electron microscope.

### Ca^2+^ sensitive CRET activities on cellular extracts and transgenic mouse tissue

Cells were prepared as previously described [14]. For green/blue photon ratio determinations, cellular extracts and purified mitochondrial fractions from transgenic mice (prepared as described above), were incubated with 5 µM coelenterazine (Interchim, Montluçon, France). Equal aliquots of each sample were placed into wells of a 96-well plate. Light was recorded for 5 sec (1 sec integration) and then a 50 mM CaCl_2_ solution was injected into the well and recording was continued for a further 30 sec. Light emitted through short band-pass filters ‘blue’ (460/30 nm) and ‘green’ (515/20 nm) was detected in independent experiments using a luminometer (Mithras LB940, Berthold Technologies, Germany). The ratio of light detected (RLU, calculated as the average for the first 5 sec after injection) through the ‘green’ to ‘blue’ filters was then determined. All experiments were carried out at room temperature.

### Preparation of the coelenterazine substrate

Coelenterazine (Interchim, France) was dissolved in ethanol to a stock concentration of 10 mM and stored at −20°C. Care was taken to protect the substrate from light and oxygen. The stock solution was then diluted in sodium phosphate buffer immediately before tail-vein (i.v.) for adults or intra-peritoneal (*i.p*) injection for neonates. Coelenterazine was injected at 2–4 mg/kg mouse, in a volume of 100–150 µl for adults and 20–40 µl for neonates.

### Preparation of brain slices and *in vitro* detection of Ca^2+^-activities

0–2 day old mouse pups were decapitated and brains were rapidly removed. After removal of the cerebellum, the brain was placed on a small platform and immersed into ice-cold oxygenated (95 % O_2_/5 % CO_2_) artificial cerebrospinal fluid (ACSF) without added CaCl_2_, containing 124 mM NaCl, 3 mM KCl, 1.3 mM MgCl_2_, 25 mM NaHCO_3_, 1.25 mM NaHPO_4_ and 10 mM glucose. Horizontal or coronal slices (400 µm thick) were rapidly cut using a vibratome (Model VT-1000, Leica) and then transferred to another smaller chamber kept at room temperature and containing ACSF (as above but with 2 mM CaCl_2 _added) and also 5–10 µM coelenterazine. The chambers containing the slices were then maintained in the dark for at least 1 hour before being transferred to a slice chamber (Warner Instruments Inc. USA) for imaging and this was then placed onto an inverted microscope, where perfusion (1 ml/min) with oxygenated ACSF was continued throughout the remainder of the experiment. Slices were imaged through a 10X Plan-NEOFLUAR objective on a combined bioluminescence/fluorescence wide field microscope system as previously described [14]. The whole field of view, which was approximately 600 µm^2^ (256×256 pixels), was analysed for each experiment. Stock solutions of TTX (1 mM, Roth, Karlsruhe, Germany), D-APV (50 mM, Sigma-Aldrich), CNQX (2 mM, Sigma-Aldrich), FCCP (2 mM, Sigma-Aldrich), Piericidin A (10 µg/ml in DMSO, Sigma-Aldrich), were stored at −20°C and diluted in ACSF before addition to the perfusate.

### Sciatic nerve stimulation experiments

8–12 week old male mice with ubiquitous expression of mtGA in all cells of the muscles were used in experiments. Animals were anaesthetised by isoflurane and the fur covering the area of the hindlimb was shaved to maximize light transmission. The sciatic nerve was then isolated and a custom made platinum bipolar electrode was attached for stimulation of muscle contraction in the hindlimb. The anaesthetised mouse was then transferred to the imaging chamber and anaesthetic was continuously administered by nose cones throughout the imaging procedure. An isolated stimulator (DS2A, Digitimer Ltd, Welwyn Garden City, U.K.) was used to apply a monopolar voltage pulse (polarity was chosen to have the lowest threshold voltage), which was usually in the range of 0.5–1.5 V/0.1-5 ms. The DS2A was driven by a pulse generator (DG2, Digitimer Ltd) or a 4030 timer generator (Digitimer Ltd). Variable stimulation frequencies were applied as described in the text, with each pulse having 5 ms duration. This protocol was based on similar work previously described [8]. In studies determining the rate of photoprotein reconstitution in the muscle, CLZN was injected by tail-vein and the animal was then placed immediately inside of the imaging chamber and the acquisition was started. Trains of stimuli (2.5 s duration) at 50 Hz (5 ms pulses) were applied every 2 minutes and the amplitude of the light response was followed for up to 1.5 hours. In some studies, trains of stimuli were applied every 30 seconds. Ru360 (Calbiochem/EMD biosciences, Inc. La Jolla, CA) was dissolved in dH_2_0 and stored as aliquots in the dark at −20°C for up to 1 week before experiments. Approximately 100 µl of Ru360 (200–500 µM) was injected intramuscularly, in small aliquots (<20 µl) in 5 or 6 different locations across the hindlimb muscles.

### Non-invasive, high resolution *in vivo* detection of Ca^2+^ signals

All *in vivo* BLI images were acquired using either one of two whole-body small animal imaging systems based on a highly sensitive photon counting technique: the “Photon Imager” or the “Video Imager” (Biospace Lab, Paris, France). Both systems consist of a light tight chamber housing a third generation cooled GaAs intensified charge-coupled device (ICCD) camera (1080×1440 pixels) operating in a photon counting mode and an F 1.4 objective lens. The field of view (FOV) is either 16×12 cm or 8×6 cm depending on the platform position, yielding an “intrinsic” resolution of the camera of 100 µm (smallest FOV) or 200 µm (largest FOV). This true spatial resolution in the final image depends on light absorption and scattering in tissues and also on image smoothing, which is user-dependant. Post acquisition, spatial smoothing of the BLI data is available (i) during reconstruction of the BLI image, by a Gaussian filter with a FWHM (full width at half maximum) size of 3, 5, or 9 times the pixel size; or (ii) during reconstruction of the composite (BLI plus video) image, by a Gaussian filter with a FWHM expressed in mm. Information regarding the processing of images is given in the legend of each figure. Color scales represent photons/pixel, unless otherwise stated.

With the “Photon Imager”, video images can be taken before or after recording the bioluminescence images, for superimposition. The “Video Imager” is very similar to the “Photon Imager”, for the exception that it enables simultaneous registration of bioluminescence images and video images using infrared LEDs. The light collected by the objective lens is split by a 45° angle mirror into two beams. One of these beams is then recorded as the video signal on a CCD camera and the other beam is acquired as the BLI signal (as described above) after filtering with a short-pass filter. Both systems operate at up to 25 frames/s (40 ms exposure time) but longer integration times can be selected after acquisition for data analysis and replay.

### EMG experiments

An average of 10 electromyograms (EMG) were recorded by a ball shaped silver electrode (∼1,5 mm in diameter) covered with AgCl and positioned under the leg skin in the vicinity of the tibialis muscle while stimulating the sciatic nerve. The induced contractions were of the isotonic type, the leg being free to move. EMG signals were recorded through a NPI (Tamm, Germany) amplifier system (Ext-10C extracellular amplifier module + LPBF-01G Bessel filter–set at 200 Hz, both housed in a EPMS-07 enclosure) onto a “D.A.T.” recorder (Biologic, Claix, France) for further measurement and analysis. Measurements were performed using an Axon Instruments (now part of “Molecular Devices”, U.S.A.) TL1 acquisition system operated with Pclamp-6 software (now part of “Molecular Devices”, U.S.A.); analysis was made with the Origin-7 graph package (Northampton MA, USA). The same stimulation system as described above was used. The light intensity during muscle contractions was recorded as a function of time using 40 ms frames. Light and EMG records were synchronised by generating a dim pulse of light from a small LED located near by the animal under study in the imager, in synchrony with the trigger applied to the isolated stimulator.

### *In vivo* detection of Ca^2+^ activity in freely moving mice

1–2 day old mice expressing mtGA were injected (i.p.) with native coelenterazine (2–4 µg/g of body weight; Interchim France). After 1–2 hours, non-anaesthetised mice were imaged at room temperature (25°C) un-restrained and freely moving. Ca^2+^-induced bioluminescence was co-registered with the video recording using an acquisition rate of 25 Hz (Video Imager, Biospace Lab, Paris, France). Motor twitches and coordinated movements were also visually recorded by placing a scoring of 1 for each frame (1 frame/s) when movements were present and 0 when they were not. The resulting plot was then generated using Microsoft Excel Software. For comparison to Ca^2+^-responses, coordinated movements are defined as sustained motor activity of the limbs or head. Motor twitches are defined as phasic, rapid, and independent movements of one limb or the tail. Startles are sudden phasic contractions of the body muscles, with the simultaneous involvement of all extremities [31], [32], [38].

In additional experiments, freely moving animals were also monitored with the Photon Imager. 0–3 day old mice expressing mtGA were injected (as above) with coelenterazine and imaged 1–2 hours later at room temperature (25°C). Mouse pups were placed inside a circular cardboard barrier that was 4 cm in diameter for 30 min prior to the beginning of recordings. Recordings of mice were then undertaken for up to 1 hour. For experimental analysis, a single region of interest was drawn over the entire area within the barrier and plots with 120 ms of time integration over 1500 s were analysed. The interval between fast Ca^2+^-responses (<1 s duration) and sustained Ca^2+^-responses (>1 s) was determined. A threshold was set for Ca^2+^-responses at 30 photons/s above the mean of the baseline in subsections of the trace. The interval between sustained Ca^2+^-responses was determined by taking the time point at the start of one sustained response to the time point at the start of the next sustained response. The interval between fast Ca^2+^-transients was determined between each sleep/wake cycle (i.e. between sustained responses). The duration of sustained Ca^2+^-responses was determined as the time point at the start of the rising phase until the last visible peak or shoulder above the threshold, in order to avoid including the decay phase.

### Detection of mitochondrial Ca^2+^ signals during epileptic seizure

Mice (P6–P18) were injected (i.p.) with coelenterazine (4 µg/g). After 2 hours, kainic acid (25 mg/kg) was injected (i.p.) and the distribution of mitochondrial Ca^2+^ fluxes were monitored in the whole animal during seizure. Kainic acid (Sigma-Aldrich) was prepared by dissolving the lyophilized drug first in a drop of NaOH and then making a further dilution in PBS to the required concentration.

### Statistical analysis

Data was analyzed using Microsoft®Excel 2002. Results are given as the mean±s.e.m.

Abbreviations: [Ca^2+^], calcium concentration; [Ca^2+^]_m_, [Ca^2+^] in the mitochondrial matrix; BLI, bioluminescence imaging; ENO, early network oscillations; GFP, green fluorescent protein; GA, GFP-aequorin; CLZN, coelenterazine; CRET, chemiluminescence resonance energy transfer; hprt, Hypoxanthine Phosphorylated Ribosyl Transferase; mtGA, mitochondrially targeted GFP-aequorin; TTX, tetrodotoxin; D-APV, D-2-amino-5-phosphonovaleric acid; CNQX, 6-cyano-7-nitro-quinoxaline-2-3-dione; FCCP, carbonyl cyanide 4-(trifluoromethoxy) phenylhydrazone; SR, sarcoplasmic reticulum; YC2, second generation yellow cameleon; FOV, field of view; BRET, bioluminescence resonance energy transfer; ES, embryonic stem cells; CAG, pCMV-chicken β-actin; RLU, Relative light units; FWHM, Full-Width Half-Maximum.

## Supporting Information

Movie S1 In vivo imaging of Ca2+-induced bioluminescence in the intact mouse during hindlimb muscle contraction/relaxation induced by tetanic stimulation of the sciatic nerve (5 ms pulses at 50 Hz for a duration of 2.5 s). The sequence of bioluminescence images were superimposed over the video, which was acquired using the same protocol but in an independent experiment. The integration time for each bioluminescence image is 1 s (6 frames/s). The bioluminescence overlay has a resolution of 0.3 mm for each pixel. The light emission (photons/pixel/s) is coded in pseudocolors (0.07–0.7).
(0.74 MB MOV)

Movie S2 An example of a whole body startle. Changes in mitochondrial [Ca2+] (bioluminescence) were co-registered together with the video image in a newborn mouse (P1). The movie was recorded with the “Video Imager”. The animation represents consecutive frames recorded over approximately 1.5 s (see the corresponding light emission profile in Figure 5A). The integration time for each frame (bioluminescence & video) is 40 ms. There are 44 frames in total and the film runs approximately 3 times slower than the actual event (×0.34). Smoothing has been applied to the bioluminescent image overlay to a resolution of 5 mm. The light emission (photons/pixel) is coded in pseudocolors as outlined for Figure 5B (i).
(0.89 MB MOV)

Movie S3 An example of coordinated movement. As for Movie S2, changes in mitochondrial [Ca2+] and the video imager were recorded simultaneously. The animation represents consecutive frames recorded over approximately 5 s (see the corresponding light emission profile in Figure 5A). The integration time for each video frame is 40 ms. The bioluminescence overlay of each frame is 160 ms of integrated light. The video is made up of a series of sliding frames, each shifted by 40 ms. The actual event is seen in real time (1 X). Smoothing has been applied to the bioluminescent image overlay to a resolution of 3.5 mm. The light emission (photons/pixel) is coded in pseudocolors as outlined for Figure 5B (ii).
(2.45 MB MOV)

Movie S4 Visualization of mitochondrial Ca2+-fluxes during kainic acid-induced seizure. A 7 day old mouse pup was injected (i.p.) with coelenterazine. After 2 hours, kainic acid (25 mg/kg) was injected (i.p.) and the distribution of mitochondrial Ca2+ fluxes were monitored in the whole animal during seizure. The sequence shows whole animal Ca2+-induced bioluminescence during kainic acid induced seizure. Frames have a resolution of 300 µm for each pixel. Color scale is 0.02–1.0 photons/pixel. The integration time for each image is 30 s (6 frames/s).
(0.75 MB MOV)

Movie S5 Whole body patterns of Ca2+- induced bioluminescence approximately 50 mins after kainate-induced seizure in a 7 day old mouse pup. A video image was recorded at the beginning of the acquisition and is merged with the bioluminescence recording, which consists of 2 s frames. Smoothing has been applied to the bioluminescent image overlay to a resolution of 1 mm and the color scale is 0.01–0.1 photons/pixel. There are 50 frames in total (6 frames/s).
(1.18 MB MOV)
